# Hyperbranched Polyethylene Ionomers Containing Quaternary Ammonium Ions and Their Functionalization of Nanomaterials

**DOI:** 10.3390/nano15070525

**Published:** 2025-03-31

**Authors:** Zhibin Ye, Jalal Rahmatinejad, Bahareh Raisi, Peishuai Dai

**Affiliations:** Department of Chemical and Materials Engineering, Concordia University, Montreal, QC H3G 1M8, Canada; j_rahmat@live.concordia.ca (J.R.); bahareh.raisi@mail.concordia.ca (B.R.); peishuai.dai@mail.concordia.ca (P.D.)

**Keywords:** ionomers, polyethylene, synthesis, catalytic polymerization, nanomaterials

## Abstract

Ionomers containing a small number of ionic side groups are a unique class of polymers with some valuable properties and distinct applications. To date, commercially important ionomers are exclusively anionomers that contain covalently bonded anions and are synthesized commonly by radical polymerization. The catalytic synthesis of polyethylene-based cationomers is challenging, while it is attractive due to the low cost of ethylene stocks and less stringent polymerization conditions, along with their desirable properties and broadened scope of commercial applications. Advances in catalyst technology—specifically, Pd-diimine catalysts—have recently enabled the synthesis of a class of hyperbranched polyethylene cationomers that are designed to contain quaternary ammonium cations. With their unique hydrophobic hyperbranched polyethylene backbone, this class of ionomers enables the successful functionalization of negatively surface-charged nanomaterials, thus improving the processing and application of the latter. This review summarizes the developments of this class of ionomers, including their synthesis, properties, and functionalization of various nanomaterials.

## 1. Introduction to Ionomers and Their Synthesis

Ionomers are a unique family of polymers designed with small fractions (typically <15 mol%) of ionic groups that are commonly covalently bonded to a hydrophobic polymer backbone as side groups. Depending on the types of ionic groups, ionomers can be classified into anionomers containing covalently bonded anions (e.g., carboxylate, sulfonate, and phosphate) and cationomers bound with cations (e.g., quaternary amine, pyridinium, and imidazolium). Due to the strong interactions of polar ionic groups and their thermodynamic incompatibility with the nonpolar polymer backbone, ionic groups tend to aggregate to form microphase separation in the solid state, with nanoscale ionic aggregates/clusters dispersed within a nonpolar polymer matrix. The formation of this microphase separation morphology yields some valuable polymer properties, including ion conductivity, strength, toughness, surface properties, barrier properties, adhesion, etc. In consequence, ionomers find extensive applications in some important technological areas, such as ion transport membranes for fuel cells and electrolyzers, binders in batteries and fuel cells, thermoplastic elastomers, adhesives, etc. [1,2,3].

Ionomers are typically synthesized by either the direct copolymerization of vinyl monomers with ionic comonomers containing an ionic group or the postpolymerization ionization of electrically neutral polymers, with the former preferred due to the better tunability in ionomer microstructures. With the direct polymerization approach, the polymerization mechanism should be amenable to ionic monomers containing polar ionic groups, including free radical polymerization and controlled/“living” radical polymerization. Meanwhile, polymerization mechanisms where active species can be poisoned by ionic groups should be avoided, such as metal-catalyzed polymerization employing metal catalysts that are sensitive to polar ionic groups [4].

To date, a variety of ionomers with different combinations of polymer backbone types and bound ion types have been developed, with some of them in widespread commercial applications. Some well-known examples are sulfonated perfluorocarbon (Nafion), polyethylene-based ionomers (Surlyn), and sulfonated polystyrene [1,2,3,5]. All of them are anionomers bound with carboxylate or sulfonate anions and are exclusively synthesized by radical polymerization. In contrast, commercial cationomers bearing positively charged cations are relatively less common, while they are important in some distinct application areas where negatively charged anionomers cannot replace them—for example, anion exchange membranes in alkaline fuel cells [5].

**Scheme 1:**
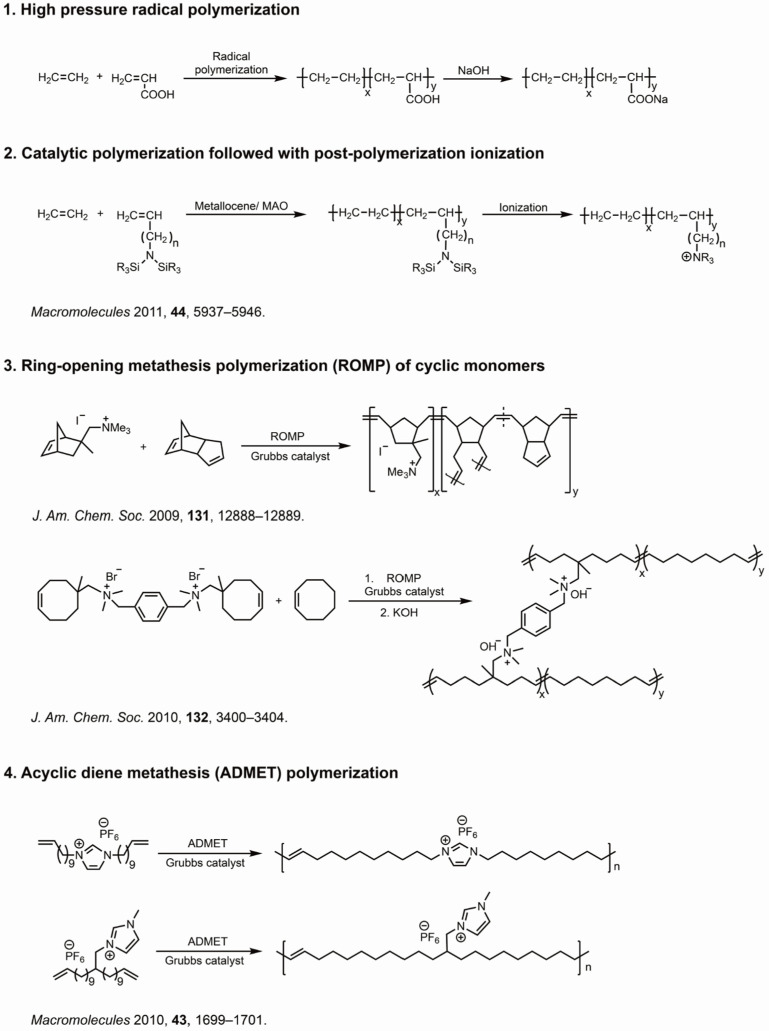
Some common strategies for the synthesis of polyolefin ionomers.

Among the various ionomers, polyethylene-based ionomers are especially attractive due to the low cost of ethylene and their valuable properties, including the outstanding thermal/chemical stability of the semicrystalline polyethylene backbones, superior mechanical properties, and thermoplastic-like processability. Polyethylene-based Surlyn ionomers are commercially produced via the radical copolymerization of ethylene with (meth)acrylic acid, followed by postpolymerization neutralization with a base [3]. It is known that the radical processes for ethylene-based monomers require demanding high-temperature and high-pressure reaction conditions, which are relatively energy-intensive and costly. On the other hand, the catalytic coordination polymerization processes commonly used for the production of the majority of commercial polyethylene and polyolefin grades need significantly less stringent conditions with considerably lower temperatures and pressures; thus, they are much preferred for the production of polyethylene ionomers. However, the direct catalytic coordinative copolymerization of ethylene with ionic comonomers is prohibitively challenging, although it is desirable. This is because most transition metal catalysts (Ziegler−Natta or metallocene catalysts) are often highly sensitive to and easily poisoned by heteroatoms present in ionic comonomers [4]. In consequence, polyethylene and other polyolefin-based ionomers have been synthesized by direct radical polymerization or by postpolymerization ionization following coordinative polymerization [6,7,8,9,10,11]. Alternatively, coordinative metathesis polymerization with the use of Ru catalysts that are tolerant of polar ionic groups has also been reported for polyolefin ionomer synthesis by direct polymerization, but with the requirement of expensive special monomers (e.g., cyclic olefins and acyclic dienes) [5,12,13,14,15]. Scheme 1 shows these existing common strategies for the synthesis of polyolefin ionomers.

Designing polyethylene ionomers—particularly cationomers, given their scarcity—by direct catalytic coordinative polymerization from an ethylene stock is thus highly desirable. Such polyethylene cationomers are poised to become new polyethylene ionomer grades, with promise for emerging technological applications—for example, the functionalization of various negatively surface-charged nanomaterials for applications in broad areas. The key to the success of this goal is the discovery of efficient transition metal catalysts that can facilitate the direct catalytic polymerization of ethylene and ionic comonomers for their synthesis.

## 2. Catalytic Synthesis of Hyperbranched Polyethylene Ionomers Containing Quaternary Ammonium Ions

**Scheme 2:**
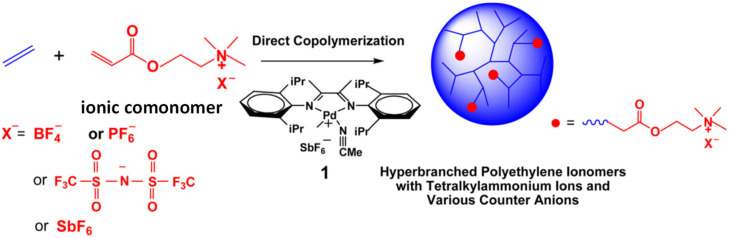
Synthesis of hyperbranched polyethylene ionomers containing quaternary ammonium ions with various counter anions. Adapted from Ref. [4] with permission from the American Chemical Society.

Tackling the above challenge, our group reported, in 2015, the first catalytic synthesis of a class of hyperbranched polyethylene ionomers designed with quaternary ammonium cations by the direct coordinative copolymerization of ethylene with an acrylate ionic comonomer (see Scheme 2) [4]. The acrylate ionic comonomer has a quaternary ammonium ion with a complementary weakly coordinating counter anion (BF_4_^−^, PF_6_^−^, SbF_6_^−^, Tf_2_N^−^). The key to this synthesis is the use of a Pd-diimine catalyst for the polymerization (**1** in Scheme 2). First discovered by Brookhart et al. in 1995 [16], Pd-diimine catalysts, along with their Ni analogs, represent a generation of single-site late transition metal catalysts with unprecedented catalytic performance compared to early transition metal-based Ziegler–Natta and metallocene catalysts [17].

Relative to early transition metal catalysts, Pd-diimine catalysts are much less sensitive to functional groups due to the known low oxophilicity of Pd as a late transition metal. It has been demonstrated that Pd-diimine catalysts enable the copolymerization of ethylene with a variety of functionalized vinyl monomers bearing different oxygen-containing functionalities (such as ester, ketone, epoxide, carbohydrate, etc.) [18,19]. In particular, these catalysts are known to copolymerize ethylene with acrylate-type comonomers. This unique feature of Pd-diimine catalysts enables, for the first time, the synthesis of functionalized polyethylenes by direct copolymerization [18]. Their low oxophilicity also allows them to facilitate polymerization even in highly polar solvents without poisoning—for example, water, acetone, etc. [20]. In the synthesis of such ionomers concerned herein, acetone is intentionally used as the typical polymerization solvent in order to solubilize the highly polar ionic comonomers, which do not dissolve in nonpolar or low-polarity solvents [4].

Another distinct feature of Pd-diimine catalysts is their unprecedented chain walking mechanism [16,21]. During chain propagation, the catalytic metal center, at the ethylene-dissociated state, can undergo isomerization or chain walking through a sequential mechanistic process comprising β-H elimination (yielding an olefin hydride complex), the bond rotation of the trapped olefin, and reinsertion to render a methyl-branched alkyl complex. Further isomerization through the same process leads to the alkyl complex with a longer branch. Subsequent ethylene trapping and insertion with the alkyl complex produces a polyethylene sequence with the corresponding branch [16,21]. Pd-diimine catalysts are noted for their fast chain walking, rendering extensively branched polyethylenes with high branch densities. The chain walking distance can be tuned by adjusting the competition between chain walking and chain propagation [21,22,23]. Typically, the hyperbranched/dendritic topology results at a relatively low ethylene pressure (e.g., 1 atm), while the topology is linearized with an increase in the ethylene pressure (e.g., 27.2 atm) [22]. This marks a unique difference relative to early transition metal catalysts, which require the addition of olefin comonomers in order to generate branched polyethylenes. Additionally, the catalysts are also noted for their outstanding capability of facilitating the “living” polymerization of ethylene and olefins under certain conditions, which enables the design of polyethylenes of other complex architectures (block, star, gradient, gradient–block, etc.) [24,25,26,27,28,29,30,31].

Herein, in the synthesis of hyperbranched polyethylene ionomers, the Pd-diimine catalyst well tolerates both the acrylate and quaternary ammonium ionic groups without experiencing apparent poisoning. However, the counter anion of the ionic group has pronounced effects on the polymerization. The copolymerization proceeds well with the ionic comonomers bearing BF_4_^−^, PF_6_^−^, SbF_6_^−^, and Tf_2_N^−^, respectively, as the counter anion, rendering successfully hyperbranched polyethylene ionomers at the ethylene pressure of 1 atm and 25 °C. However, no polymerization occurs in the case with ionic comonomers with Cl^−^ or trifluoromethanesulfonate (TfO^−^) counter anions [4]. This distinct difference arises from the differences in the coordination power of the anions. While the former are very weakly coordinating anions, TfO^−^ is moderately weakly coordinating and Cl^−^ is strongly coordinating. The latter two can thus coordinate to the Pd metal center, making monomer coordination difficult and poisoning the active center. Ionomers bearing the former four counter anions (BF_4_^−^, PF_6_^−^, SbF_6_^−^, and Tf_2_N^−^) can thus be directly produced by polymerization, while those with the latter two (TfO^−^ and Cl^−^) can only be obtained indirectly from ionomers produced with one of the four former anions by anion exchange [4].

A change in the feed concentration of the ionic comonomer, while the other conditions are identical (ethylene pressure of 1 atm and 25 °C), allows the tuning of the ionic content in the ionomer. An increase in the feed concentration of the ionic comonomer with BF_4_^−^ counter anion from 0.1 to 1 M yields an increase in its content in the resulting ionomers from 0.4 to 2.3 mol%. Among the ionomers synthesized with ionic comonomers with different counter anions but at the same feed concentration (0.3 M), the ionic comonomer content increases in the anion order of PF_6_^−^ < Tf_2_N^−^ < SbF_6_^−^ < BF_4_^−^. This suggests that BF_4_^−^ is the least coordinating anion among the four, and the other three have similar but slightly higher coordinating power than BF_4_^−^ [4]. The weight-average molecular weight (*M_w_*) of the ionomers is within the range of 10–60 kg mol^−1^, along with a polydispersity index (PDI) of around 1.5–3.0. Meanwhile, *M_w_* shows a trend of decrease with an increase in the feed concentration of the ionic comonomer, along with a dependence on the anion type [4]. Regardless of the ionic comonomer type or content, the ionomers have a similar hyperbranched polyethylene backbone with a similar branching density (about 90 branches per 1000 carbons).

This unique polymerization system thus enables the direct synthesis of a class of hyperbranched polyethylene ionomers with quaternary ammonium cations at tunable content (up to about 2–3 mol%) with different counter anions (BF_4_^−^, PF_6_^−^, Tf_2_N^−^, SbF_6_^−^). Despite this, a major drawback of this synthesis is the high cost of the Pd-diimine catalyst, as well as its low polymerization activity (ethylene turnover frequency: about 20 h^−1^), which are common for this series of catalysts. It is also desirable to synthesize analogous ionomers bearing other types of cations, such as imidazolium and pyridinium. Our group has also attempted to copolymerize ethylene with 1-[2-(acryloyloxy)ethyl]-3-butyl-imidazolium tetrafluoroborate, an imidazolium-containing acrylate-type ionic liquid comonomer with BF_4_^−^ counter anion, with the polymerization system [4]. However, no polymerization occurred, likely due to catalyst poisoning by the imidazolium functionality, thus excluding the use of imidazolium-containing monomers in this polymerization system. Meanwhile, no study on copolymerization with pyridinium-containing ionic liquid comonomers has been reported yet.

To date, only catalyst **1** with the simplest diimine structure has been used for ionomer synthesis, as it is known to produce the hyperbranched polyethylene backbone. In general, diimine ligand sterics and electronics are expected to impact the branching density, topology, and molecular weight of the resulting polyethylene backbone, as well as the polymerization activity. The exact effects in this specific polymerization system for ionomer synthesis have not been investigated, although some general ligand effects are known for ethylene polymerization with this series of catalysts [17].

Subsequent to our report [4], a few other groups have also reported the direct catalytic synthesis of polyethylene ionomers with Pd-based catalysts. Chen and Dai demonstrated the synthesis of polyethylene ionomers bound with carboxylic acid groups by ethylene copolymerization with acrylic acid, allylacetic acid, or undecanoic acid, which was facilitated by Pd-diimine catalysts having bulkier diimine ligands [32]. The absence of catalyst poisoning in the polymerization was proposed to result from the existence of acidic comonomers in the dimeric form. Nevertheless, these ethylene–acrylic acid copolymers somewhat resemble anionomers produced by radical processes. In addition, Mecking et al. reported the direct catalytic synthesis of imidazolium-functionalized polyethylene cationomers by ethylene copolymerization with allyl imidazolium tetrafluoroborate with phosphinesulfonate palladium catalysts [33]. Notably, no catalyst poisoning by the imidazolium group was found, rendering cationomers with ionic content of up to 0.67 mol%. However, no further study on the properties and applications of these polyethylene cationomers was reported.

## 3. Structure and Properties of Hyperbranched Polyethylene Ionomers

The ionomers synthesized through this strategy feature a nonpolar branched polyethylene skeleton tethered with the highly polar quaternary ammonium cations. Typically, the branched skeleton has a high branching density of about 90 branches per 1000 carbons, according to ^1^H nuclear magnetic resonance (NMR) spectroscopy. Under the typical polymerization conditions (1 atm of ethylene pressure and 25 °C), the polyethylene skeleton should have the highly compact hyperbranched chain topology. According to the unique insertion chemistry of acrylate-type monomers with Pd-diimine catalysts in ethylene copolymerization, each comonomer is incorporated at a branch end and each ionic group is thus located at a branch end [4].

The incorporation of the ionic groups shows dramatic effects on some of the polymers’ physical properties. Hyperbranched polyethylene homopolymers produced under the same conditions have relatively high molecular weights (weight-average molecular weight of around 36,000 g mol^−1^) and are typically low-viscosity Newtonian oils (e.g., viscosity of around 20 Pa s) at room temperature as a result of their hyperbranched chain topology [4,22]. Due to their nonpolar nature, they are only soluble in nonpolar or low-polarity solvents, such as toluene, hexane, tetrahydrofuran, chloroform, etc., while insoluble in highly polar solvents, such as dimethylformamide (DMF) and acetone [34]. Following the incorporation of the ionic comonomer, the ionomers have reduced molecular weights (around 10,000 to 23,000 g mol^−1^) compared to homopolymers, with a negative dependence on the ionic comonomer content. Due to the presence of the highly polar ionic groups that form physical cross-linking by ionic interactions, the ionomers instead are non-transparent, non-flowable, gel-like solids at room temperature. Meanwhile, they are soluble/dispersible in highly polar solvents such as DMF, besides nonpolar or low-polarity solvents [4]. This makes them valuable for applications in the modification of nanomaterials towards their processing and storage in organic solvents, as summarized in the following section. In their dispersed states in the solvents, ionic aggregation also leads to the formation of physical cross-linking. With the possession of quaternary ammonium ions, dispersions of the ionomers in organic solvents show positive zeta potentials of around 10–50 mV, depending on the ion content [4].

The covalently tethered ionic groups show relatively weak but distinct impacts on the thermal transitions of the hyperbranched polyethylene skeleton. Hyperbranched polyethylene homopolymers typically show a glass transition (*T*_g_) centered at about −64 °C and a weak but broad melting peak over the range of about −58 to 20 °C, with a peak maximum (*T*_m_) at −17 °C and melting endotherm (Δ*H*_m_) of about 25 J g^−1^. Upon the incorporation of the ionic comonomer, a slight increase in *T*_g_ and a slight decrease in *T*_m_ are seen, along with the narrowing and weakening of the melting endotherm. These effects on the polyethylene skeleton are increasingly pronounced with an increase in the content of the ionic comonomer. However, thermal transitions relating to the aggregated ionic clusters cannot be detected with differential scanning calorimetry (DSC) due to their low content [4].

Because of the formation of physical cross-links by strong ion aggregation, the incorporation of the ionic groups often exerts dramatic effects on the flow behavior of polymers. Our group has systematically studied the melt rheological properties of this class of hyperbranched polyethylene ionomers [4,35]. In general, the melt rheology of classical ionomers has been extensively studied in the literature [36], where the rheology is usually impacted by both chain entanglement and ionic aggregation, and it is often difficult to separate the two relaxation mechanisms [37,38]. With hyperbranched polyethylene ionomers, the advantage is that the effects of chain entanglement are eliminated due to the absence of chain entanglement as a result of the hyperbranched chain topology and the relatively low molecular weights of the polyethylene skeletons. This allows us to isolate the sole effects of ionic aggregation on the flow behavior [4,35].

**Figure 1:**
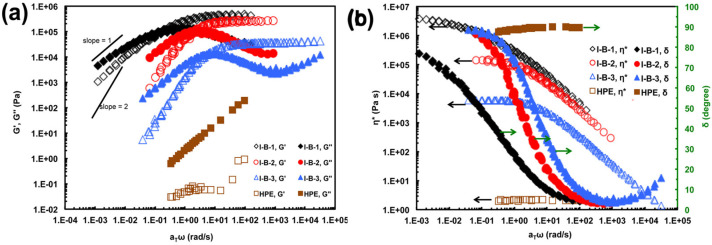
Melt rheological master curves of (**a**) dynamic moduli (*G*′ and *G*″) and (**b**) complex viscosity (η*) and phase angles (*δ*) of hyperbranched polyethylene ionomers (I-B-1, I-B-2, and I-B-3 with ion content of 2.3, 1.4, and 0.4 mol%, respectively) and polyethylene homopolymer HPE at the reference temperature of 95 °C. Adapted from Ref. [4] with permission from the American Chemical Society.

For this purpose, ionomers with different ion content but with the same counter anions have been characterized with small-amplitude dynamic oscillation to study the effects of the ion content on their melt rheological properties. Representatively, Figure 1 shows the master curves of the melt rheological properties (dynamic moduli, *G*′ and *G*″, complex viscosity, η*, and phase angle, *δ*) of a set of ionomers (I-B-1 to I-B-3) with different ion content (2.3, 1.4, and 0.4 mol%, respectively) but all with BF_4_^−^ counter anion at the reference temperature of 95 °C, along with those of nonionic hyperbranched homopolyethylene (HPE) for comparison [4]. HPE shows a Newtonian flow with a low constant η* (2.2 Pa s) and a constant *δ* of 90° across the investigated frequency range, and its *G*″ curve is well above the *G*′ curve with no crossover, confirming the absence of chain entanglement. With the ionomers, some characteristic changes are noted, including non-Newtonian flows with enormously enhanced zero-shear viscosity (*η*_0_) and shear thinning, and significantly enhanced *G*′ and *G*″ curves with a crossover point, confirming the involvement of physical cross-linking by ion aggregation. These changes become increasingly pronounced with the increase of ion content.

Other key rheological parameters have been calculated, including the flow activation energy (*E*_a_), terminal relaxation time (*τ*), and plateau modulus (*G*_N_^0^). With the increase of ion content, significant increases in *τ* and *G*_N_^0^ are noted, along with an appreciable increase in *E*_a_. In particular, *η*_0_ increases from 6.1 × 10^3^ to 1.5 × 10^5^ and to 4.4 × 10^6^ Pa s with the increase of ion content from I-B-3 to I-B-1. Relative to HPE, *η*_0_ for I-B-3 and I-B-1 is about three thousand and two million times larger, respectively [4].

**Figure 2:**
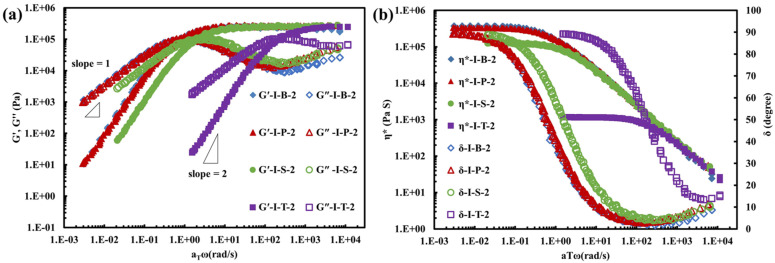
Melt rheological master curves of (**a**) dynamic moduli (*G*′ and *G*″) and (**b**) complex viscosity (η*) and phase angles (*δ*) for I-#-2 set of ionomers at the reference temperature of 75 °C. Adapted from Ref. [35] with permission from Wiley.

Our group has also investigated the effects of the counter anion size on the rheological properties of ionomers [35]. Ionomers with fixed ion contents but with different counter anions (BF_4_^−^, PF_6_^−^, SbF_6_^−^, and Tf_2_N^−^) were synthesized for this investigation. While the one with BF_4_^−^ was synthesized directly by Pd-diimine-catalyzed polymerization, the others were obtained from it by anion exchange with corresponding salts, which warranted their identical chain structures and ion contents. These counter anions show increasing sizes in the order of BF_4_^−^ < PF_6_^−^ < SbF_6_^−^ < Tf_2_N^−^, which is expected to render weakened interactions with the quaternary ammonium ions in this order. Figure 2 shows the master curves of the rheological properties (*G*′, *G*″, η*, and *δ*) of the set of ionomers with ion content of 1.1 mol% (I-B-2, I-P-2, I-S-2, and I-T-2 with BF_4_^−^, PF_6_^−^, SbF_6_^−^, and Tf_2_N^−^, respectively) at 75 °C [35].

The different counter anions show some distinct effects on the rheological properties. With each ionomer, one can see a relaxation peak in *G*″ and a rubbery plateau in *G*′, which confirm the presence of ion aggregates. The relaxation peak shows a clear shift towards the lower frequency (278, 3.3, 1.0, and 1.2 rad s^−1^) with the decrease of the anion size from Tf_2_N^−^ to SbF_6_^−^ and to BF_4_^−^/PF_6_^−^, suggesting an increasing ionic interaction force with the decreasing anion size. However, the breadth of the rubbery plateau shows an increasing trend with the decrease in the counter anion size. The crossover of the *G*′ and *G*″ curves is reduced by over two decades with the decrease in the counter anion size from Tf_2_N^−^ to BF_4_^−^/PF_6_^−^. Concomitantly, *η*_0_ increases by over 300 times from 1.2 × 10^3^ to 3.7 × 10^5^ Pa s with the decrease in the counter anion size from Tf_2_N^−^ to BF_4_^−^. *τ* increases by three orders of magnitude from 0.0076 s for I-T-2 to 3 s for I-B-2. A trend of an increase in *E*_a_ from 85 to 112 kJ mol^−1^ was also noted with the decrease in the counter anion size from Tf_2_N^−^ to BF_4_^−^. These changes in the rheological properties reflect the strengthened ionic interactions with the decrease in the counter anion size. However, *G*_N_^0^ shows no dependency on the counter anion size, while it is strongly related to the ion content [35].

## 4. Functionalization of Nanomaterials with Hyperbranched Polyethylene Ionomers for Targeted Applications

Featuring unique chain structures with a nonpolar hyperbranched polyethylene skeleton functionalized with positively charged quaternary ammonium ions, hyperbranched polyethylene ionomers show some distinct advantages for the functionalization and modification of various nanomaterials towards their processing, storage, and application [39,40,41,42,43]. The majority of nanomaterials, based on metal oxides, metallic nanostructures, metal sulfides, carbon, etc., are often synthesized/processed and stored in aqueous systems and typically bear negative surface charges under neutral or near-neutral pH conditions. These nanomaterials are generally compatible with and stably dispersible in aqueous phases or selected highly polar organic solvents/matrices. However, they are often incompatible/unstable in nonpolar or low-polarity organic solvents/matrices and tend to easily aggregate/precipitate, leading to the collapse of their nanostructures and, in turn, drastically compromised properties. This significantly restricts their application in a broader scope, where their dispersion/compatibilization with nonpolar or low-polarity media is required. The hyperbranched polyethylene cationomers considered herein are distinctly suited for the modification of these nanomaterials. They are expected to bind to their negatively charged surfaces, with their quaternary ammonium ions serving as anchors through strong ionic interactions. The presence of surface-bound ionomers is reasoned to drastically change the surface properties of the nanomaterials, from highly polar hydrophilic to nonpolar hydrophobic, due to the presence of the nonpolar hyperbranched polyethylene coating. This, in turn, renders modified nanomaterials with the desired solubility/dispersion in nonpolar or low-polarity solvents/matrices for some targeted applications due to the unique hyperbranched polyethylene backbone [39,40,41,42,43]. If the polyethylene ionomers have simply a branched but linear architecture, the solubility/dispersion of the ionomers and their modified nanomaterials in such media would be restricted, with insufficient performance properties for such applications. Meanwhile, anionomers with negative charges would not be suitable due to the typically negatively charged surfaces of most nanomaterials. In this context, our group has investigated the modification of the following nanomaterials with hyperbranched polyethylene ionomers and explored the properties and applications of the resulting modified nanomaterials.

### 4.1. Cellulose Nanocrystals

Produced from abundant and renewable biosources, cellulose nanocrystals (CNCs) are a sustainable class of nanomaterials that have attracted significant interest [44]. Featuring a rod-like shape with a diameter between 5 and 15 nm and a length of around 100 to 300 nm, CNCs are noted for their high elastic moduli (>100 GPa) and large specific surface area (a few hundred m^2^/g), with strong potential as reinforcing bionanofillers for high-performance polymer nanocomposites [44]. CNCs are generally produced as stable aqueous dispersions by the hydrolysis of cellulose fibers in the presence of sulfuric acid. Their surfaces contain negatively charged sulfate groups and are thus highly polar [45]. Their dispersions in nonpolar solvents/matrices are poor. Their applications are thus restricted generally to aqueous systems. For broadened applications, surface modification of CNCs is required to facilitate their compatibility with nonpolar systems [44].

**Scheme 3:**
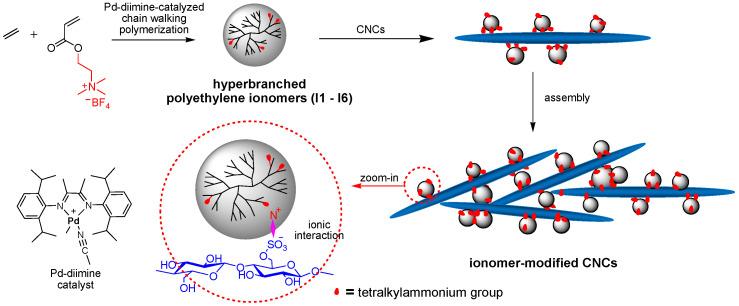
Schematic modification of CNCs with hyperbranched polyethylene ionomers. Reproduced from Ref. [39] with permission from the American Chemical Society.

Our group has demonstrated the surface modification of CNCs with the use of quaternary ammonium-containing hyperbranched polyethylene ionomers (see Scheme 3) [39]. The modification is conveniently accomplished by the simple dropwise addition of an aqueous CNC dispersion into a dilute solution of the ionomer in THF at a prescribed concentration under sonication. A study on the effects of the ion content of the ionomer and the ionomer/CNC feed mass ratio on the modification was performed. It was found that increasing the ionomer/CNC feed ratio (from 0.5 to 4) for a given ionomer with an ion content of 0.7 mol% led to a significant increase in the ionomer mass content (8 to 35 wt%) in the modified CNCs. At a fixed ionomer/CNC feed ratio of 2, the increase in ion content in the ionomer from 0.2 to 0.7 mol% led to enhanced ionomer binding from 8 to 27 wt% due to the increase in binding sites for ionic complexation with the CNCs. However, the effect leveled off with a further increase in ion content [39].

**Figure 3:**
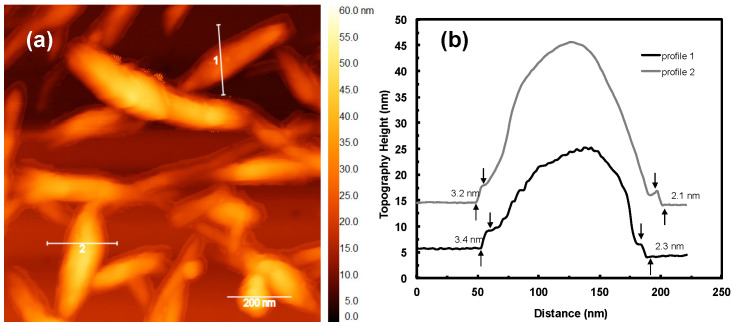
AFM height image of a typical modified CNC sample and height profiles across two shapes in the image. Reproduced from Ref. [39] with permission from the American Chemical Society.

Figure 3 shows an atom-force microscopy (AFM) height image of a typical modified CNC sample and height profiles across two shapes in the image. The ionomer modification induces the self-assembled aggregation of individual CNC rods and rod aggregates due to the “cross-linking” effect of multidentate ionomers. The presence of the ionomer coating is seen at the edge of each shape in the AFM image and height profiles [39].

Relative to the negative ζ potential of −27 mV of the original CNCs, the ionomer-modified CNCs showed increased ζ potential from 17 to 30 mV, depending on the ion content in the ionomers. With the exception of those with insufficient ionomer coating (ionomer content < 10 wt%), the resulting ionomer-modified CNCs could be stably dispersed in nonpolar or low-polarity organic solvents, including chloroform, toluene, and THF, at dilute concentrations (about 10 mg mL^−1^), but not in high-polarity solvents (methanol, acetone, etc.) [39].

Dispersions of modified CNCs in THF at a relatively high concentration within about 25–75 mg mL^−1^ were found to exhibit distinct thixotropic rheological behavior. When stable, the dispersions existed as white-colored non-flowable organo-gels, indicating the presence of gel network structures as a result of the high positive surface charge of the particles of modified CNCs. After shaking, the gels become flowable fluids, suggesting the collapse of the network structures under shear. Rheological characterization by small-amplitude dynamic oscillation and steady shear measurements were performed, confirming the thixotropic behavior of the organo-gels. It was also found that the ion content of the ionomers played an important role in the formation of the gel network. Only the dispersions of modified CNCs prepared with ionomers with ion content of >0.7 mol% showed gel formation due to the sufficient surface charge [39]. The unique thixotropic behavior facilitates the potential applications of ionomer-modified CNCs as rheological agents for paints, inks, and other products where thixotropic properties are desired.

The ionomer-modified CNCs were also investigated for their dispersion and reinforcing performance as fillers in a hydrophobic ethylene–olefin copolymer elastomer as the polymer matrix. The rheological characterization of the composites showed significant reinforcement effects at the CNC loading of 10 wt% upon the addition of the modified CNCs, as well as the formation of percolated filler networks. The reinforcement effects were significantly strengthened compared to the composites with bare unmodified CNCs. Meanwhile, with cetyltrimethylammonium bromide (CTAB)-modified CNCs, no percolated filler network was found at the same CNC loading. This comparison suggests that ionomer modification renders the fillers more compatible with the EOC matrix, which improves filler dispersion, minimizes filler aggregation, and increases the filler–matrix interface. Tensile mechanical tests further showed that composites with modified CNCs had higher elongation at break than those with unmodified and CTAB-modified CNCs at identical CNC loading, further confirming the benefits of ionomer modification in improving the composite’s performance by enhancing the filler dispersion in the nonpolar EOC matrix [39].

### 4.2. Gold Nanorods

Gold nanorods (GNRs) are a distinct class of anisotropic metallic nanoparticles with valuable optical and photothermal properties and have attracted substantial research interest for applications in various areas [46]. GNRs are commonly synthesized as aqueous dispersions through the seed-mediated growth methodology, which requires CTAB as both the surface-stabilizing ligand and shape-directing agent [47]. The as-prepared GNRs are coated non-covalently with a CTAB bilayer, where the cationic quaternary ammonium head groups in the inner layer bind strongly to the rod surface and those in the outer layer protrude out to ensure the stability of the GNRs in the aqueous solution [48]. The dynamic exchange of CTAB molecules occurs between the rod surface and solution, and a minimum concentration of free CTAB is required in the aqueous solution to maintain the stability of the CTAB-coated GNRs [49]. Although stable in aqueous solutions containing sufficient free CTAB, CTAB-coated GNRs quickly aggregate in most organic solvents due to the destabilization of the CTAB bilayer. The surface functionalization of GNRs by displacing CTAB with another appropriate ligand via ligand exchange is highly desired to enable their solubility/dispersibility in broader media beyond aqueous systems and to provide added functionalities for broadened application [40].

**Scheme 4:**
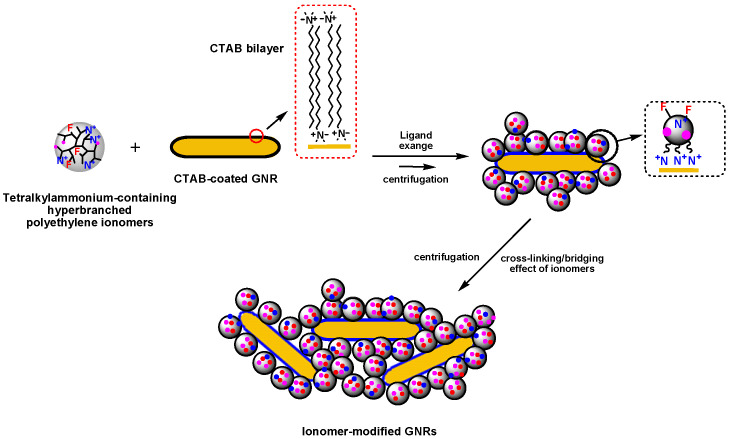
Schematic modification of GNRs with hyperbranched polyethylene ionomers. Reproduced from Ref. [40] with permission from the Royal Society of Chemistry.

**Figure 4:**
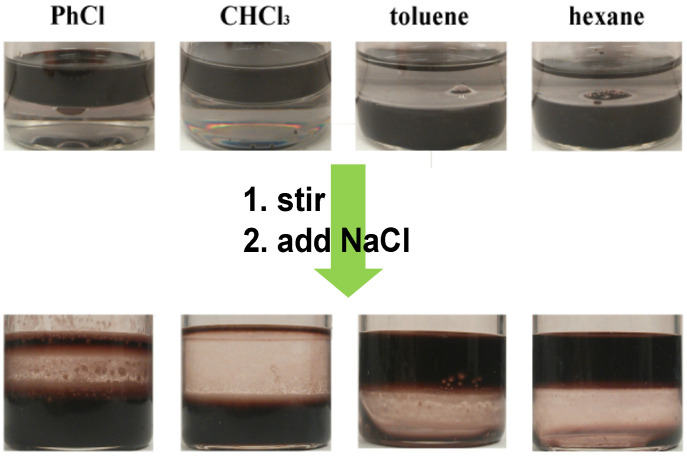
Photos showing phase transfer of CTAB-coated GNRs from aqueous to four nonpolar or low-polarity organic phases (chlorobenzene, chloroform, toluene, and hexane) containing a hyperbranched polyethylene ionomer. Adapted from Ref. [40] with permission from the Royal Society of Chemistry.

Our group has demonstrated the use of quaternary ammonium-containing hyperbranched polyethylene ionomers with BF_4_^−^ counter anion as a multidentate macromolecular surface ligand for the surface modification of GNRs (average rod diameter, 7.4 nm; average aspect ratios, 4.7 and 6.0, respectively). Functioning as the macromolecular analog of CTAB with a quaternary ammonium head group, the ionomers were reasoned to displace CTAB and bind to the surfaces of GNRs through the covalently tethered quaternary ammonium ions, which in turn modified the surface properties of the GNRs to enable their dispersibility in nonpolar/low-polarity solvents/media (see Scheme 4). This ligand exchange mechanism was verified to be the case. The convenient phase transfer of GNRs was observed from the aqueous phase to various water-immiscible nonpolar or low-polarity phases (e.g., toluene, hexane, chloroform, and chlorobenzene) containing a hyperbranched polyethylene ionomer with ion content of 1.2 mol%, following thorough mixing and the subsequent addition of a small amount of NaCl (see Figure 4) [40].

A direct dropping method was further applied to prepare CTAB-free, ionomer-modified GNRs with the use of a range of hyperbranched polyethylene ionomers designed with different ion content (about 0.2–1.8 mol%), as well as pyrene groups (0.07 to 0.63 mol%) as fluorescent labels. In this direct dropping method, an aqueous dispersion of GNRs was dropped directly, under stirring, into an ionomer solution in THF. The resulting ionomer-modified GNRs were subjected to multiple rounds of washing cycles under high-speed centrifugation (11,176 g; 20 min) and redissolution in fresh THF. The effects of the ion content of the ionomers and the ionomer dosage in terms of the feed mass ratio of ionomer to gold dosage [(m_ionomer_/m_Au_)_0_] were investigated. An increase in the dosage of a given ionomer led to an increase in the ionomer content in the modified GNRs. An increase in the ion content of the ionomer caused a decrease in the ionomer content. At a sufficient ionomer dosage [(m_ionomer_/m_Au_)_0_ > ca. 0.9], the ionomer-modified GNRs survived the multiple cycles of high-speed centrifugation without aggregation, confirming their high stability. The composition of surface ligands on one ionomer-modified GNR sample was analyzed with ^1^H NMR spectroscopy following the complete digestion of the gold core with NaCN, which showed the sole presence of the ionomer and the absence of CTAB within the detection limit [40].

**Figure 5:**
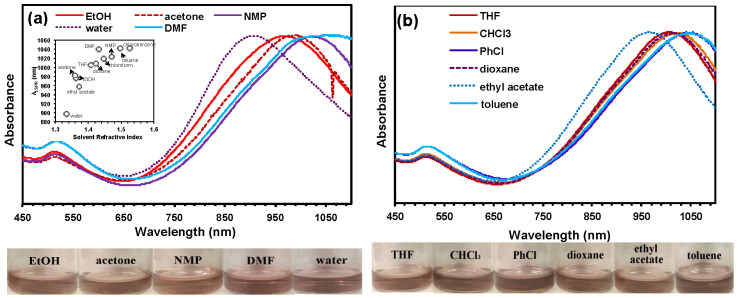
Photographs and UV–vis spectra of GNRs modified with an OEG-containing ionomer in water and ten organic solvents. Reproduced from Ref. [40] with permission from the Royal Society of Chemistry.

This ionomer modification methodology was further expanded by designing and applying amphiphilic hyperbranched polyethylene ionomers containing hydrophilic oligo(ethylene glycol) (OEG) side chains (ion content, 1.7 mol%). For this purpose, an acrylate monomer with a short OEG block was used as an additional comonomer in chain walking polymerization, aside from the ionic comonomer. The resulting modified GNRs showed dispersibility in water and ten organic solvents, including nonpolar or low-polarity ones (chlorobenzene, toluene, THF, ethyl acetate, dioxane, chloroform) and high-polarity ones (acetone, ethanol, *N*-methyl-2-pyrrolidone, DMF), confirming their amphiphilic nature (see Figure 5). The UV–vis spectra of the modified GNRs in different solvents (see Figure 5) showed similar shapes with negligible peak shifts compared to those of the original CTAB-coated GNRs, confirming the good stability of the modified GNRs and the absence of irreversible aggregation. All dispersions were stable over a period of 3 months, with no non-redispersible precipitates. Containing both hydrophobic polyethylene and hydrophilic OEG segments in the ionomer shell, the amphiphilic modified GNRs were also demonstrated in a proof-of-concept experiment, showing potential as nanoscale carriers for Nile Red as a hydrophobic guest species. This sheds light on the potential design of amphiphilic ionomer-modified GNRs as unique multifunctional drug carriers with photothermal GNR cores and fluorescent labels for cancer therapeutics [40].

### 4.3. Ti_3_C_2_T_x_ MXene

MXenes are a class of two-dimensional (2D) transition metal carbide/nitride nanosheets with the general formula M_n+1_X_n_T_x_, where M stands for the transition metal (such as Ti, Mo, etc.), X is carbon/nitrogen, and T corresponds to the surface termination moiety (such as, O, OH, or F, etc.) [50]. MXenes are commonly synthesized from the parent MAX phase with a corresponding formula of M_n+1_AX_n_ by the elimination of the “A” interlayer element (i.e., the element in groups 13 and 14 in the periodic table, such as Al). Among the numerous MXenes, Ti_3_C_2_T_x_ was the first to be discovered and is the most extensively studied one. Featuring some distinct properties, such as high electrical conductivity, mechanical properties, and hydrophilic surfaces with a strong negative charge, MXenes have found applications in various fields, including electrochemical energy storage, electromagnetics, and catalysis [51].

MXenes are commonly produced and primarily used in aqueous dispersions. They have also been reported to be dispersible in a few polar solvents with colloidal stability. However, they are unstable in nonpolar or low-polarity solvents, restricting their use in applications that require such dispersions [52]. Meanwhile, MXenes are prone to oxidation, particularly in aqueous systems, limiting their performance and storage lifetimes [53]. Their storage in nonpolar or low-polarity organic solvents is a possible solution as the contact with water and oxygen can be potentially minimized with MXene dispersions in these solvents.

**Scheme 5:**
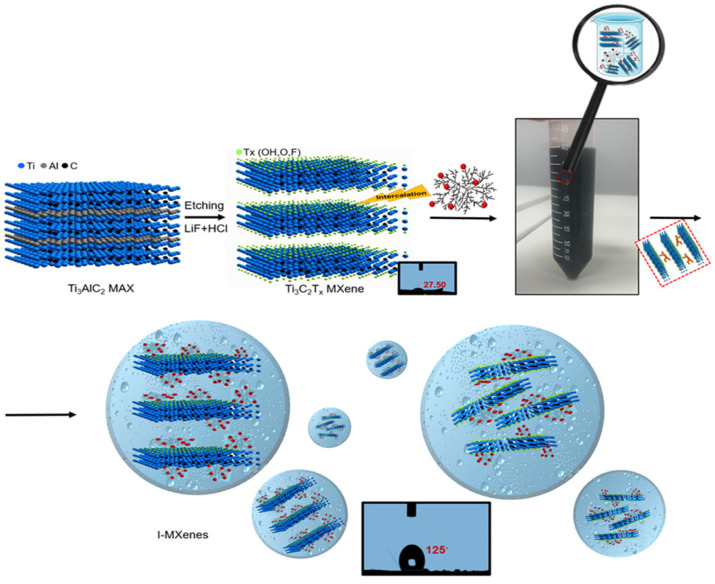
Schematic intercalation of hyperbranched polyethylene ionomers with hydrophilic MXene nanosheets to form modified MXenes. Adapted from Ref. [41] with permission from the Royal Society of Chemistry.

Our group has systematically investigated the modification of a multi-layered Ti_3_C_2_T_x_ MXene with a range of hyperbranched polyethylene ionomers containing different quantities of quaternary ammonium ions (0.31–2.87 mol%) with BF_4_^−^ counter anion [41]. For the modification, the multi-layered MXene dispersion in DMF was added dropwise to a dispersion of each ionomer in THF at a prescribed concentration under sonication. With the highly negatively charged surfaces (ζ potential of −38 mV) of Ti_3_C_2_T_x_ MXene nanosheets, the positively charged ionomers were found to bind to the surface through ionic interactions and intercalate between the MXene nanosheets, leading to the delamination of the nanosheets (see Scheme 5). X-ray diffraction (XRD) clearly verified the delamination of the MXene nanosheets upon ionomer intercalation, where the (002) peak characteristic of the interlayer distance moved from 8.15° (corresponding interlayer distance: 10.8 Å) for the unmodified MXene to as low as 1.6° (interlayer distance: 50 Å) for the ionomer-modified MXenes, accompanied by the broadening of peak width. With an increase in the ionomer/MXene feed mass ratio from 1 to 6 for the same ionomer with ion content of 1.03 mol%, the ionomer content in the modified MXenes increased from 8.7 wt% to 28.7 wt%. Meanwhile, with an increase in the ion content from 0.31 to 2.87 mol% at a given ionomer/MXene feed mass ratio of 2, the ionomer content in the modified MXenes increased from 1.8 wt% to 29 wt%, accompanied by the gradual downshift and broadening of the (002) peak [41].

The ionomer modification markedly changed surface properties of the MXene nanosheets. A film of unmodified MXene was hydrophilic, with a water contact angle of 27.5°. In contrast, a film of a modified MXene (I-MXene-4-2) with ionomer content of 18.1 wt% was found to be highly hydrophobic, with a high water contact angle of 125° due to the presence of a hydrophobic polyethylene coating. This, in turn, enabled the dispersibility of the modified MXene in various solvents. In the study, the unmodified MXene showed stable dispersibility only in selected polar solvents, including water, methanol, ethanol, acetonitrile, DMF, and acetone, as well as one low-polarity solvent (ethyl acetate). The modified MXene (I-MXene-4-2) instead showed stable dispersion in several nonpolar (including hexane, cyclohexane, toluene, xylene, benzene) and low-polarity solvents (including THF, chloroform, ethyl acetate), as well as in four polar solvents (acetonitrile, DMSO, DMF, acetone), even after 100 days. In particular, the THF dispersion of I-MXene-4-2 at a concentration as high as 31.1 mg mL^−1^ was achieved [41].

The ionomer modification also significantly improved the oxidation stability of the MXene. In open air, the aqueous dispersion of unmodified MXene was quickly oxidized within 15 days, with the formation of rutile TiO_2_, indicative of its poor oxidation stability. In contrast, a modified MXene (I-MXene-4-2) in an aqueous dispersion was found intact after 100 days under open air, with no sign of the formation of TiO_2_. Clearly, the presence of the tightly surface-bound hydrophobic hyperbranched polyethylene protected the MXene well from oxidation. This ionomer modification strategy thus facilitates the processing, handling, and storage of MXenes in nonpolar/low-polarity solvents, which significantly expands their applications [41].

In the application aspect, the ionomer-modified, delaminated, few-layered Ti_3_C_2_T_x_ MXene was demonstrated by our group to show significantly improved electrochemical performance, relative to the unmodified precursor, as a cathode material for magnesium-ion batteries (MgIB) [42]. As a type of post-lithium-ion battery, MgIBs, with a magnesium metal anode, show notable advantages, including the high volumetric theoretical capacity of the magnesium metal anode (86% higher than that of lithium metal), its high safety with significantly reduced dendrite formation, and the high natural abundance of magnesium relative to lithium [54]. Due to the divalent nature of Mg^2+^ ions, which often causes diffusion problems within common solid-state electrode materials due to strong Coulombic interactions, the development of matching cathode materials facilitating fast Mg^2+^ insertion/extraction is challenging [55]. As a class of 2D nanomaterials with layered structures, MXenes have received significant interest as cathode materials for MgIBs. Nevertheless, the insufficient interlayer spacing and restacking of the 2D nanosheets often lead to strong Coulombic interactions, hindered ion diffusion, and restricted ion accessibility. It has been shown that stacking can reduce ion diffusion by 8–20 orders of magnitude through the coordination of ions with surface oxygen atoms on MXenes [56]. The Ti_3_C_2_T_x_ MXene was reported to be unsuitable for Mg cathodes, with prohibitively slow diffusion [56]. In this regard, ionomer modification not only modifies the surfaces of nanosheets, but also prevents the nanosheets from restacking, leading to enhanced ion transport and thus improved electrochemical performance [42].

In the study [42], a delaminated Ti_3_C_2_T_x_ MXene (DL-MXene) with few-layered structures was prepared by the delamination of a multi-layered MXene (ML-MXene) through ultrasonication, followed by separation via centrifugation. The use of the DL-MXene, instead of the ML-MXene, was found to be critical due to the relatively widened ion transport passage as a result of its few-layer structure. The DL-MXene was modified with a hyperbranched polyethylene ionomer with quaternary ammonium ion content of 2.87 mol% at an ionomer/DL-MXene feed mass ratio of 2, providing the ionomer-modified sample, I@DL-MXene-5-2, with ionomer content of 38 wt%. According to the downshift of the (002) peak in XRD, I@DL-MXene-5-2 showed an enlarged interlayer distance of 1.9 nm, relative to 1.3 nm found with the unmodified DL-MXene. Under transmission electron microscopy (TEM), 2D nanosheets with significantly enlarged interplanar spacing of up to 8.6 nm were observed. It also had a high ζ potential of about 60 mV, compared to −53 mV for the DL-MXene. The results confirmed the efficient intercalation of the ionomer and exfoliation of the nanosheets, which led to an enlarged space and widened pathways for the diffusion of Mg^2+^ electrolytes. According to X-ray photoelectron (XPS) spectroscopy, I@DL-MXene-5-2 had an appreciably lowered F-element intensity relative to other elements (Ti, O) compared to the DL-MXene, indicating modified surface functionalities with reduced F^−^ ions upon ionomer binding. This may also contribute to a reduction in the Coulombic interaction of Mg^2+^ ions with the surface and thus improved electrolyte diffusion [42].

**Figure 6:**
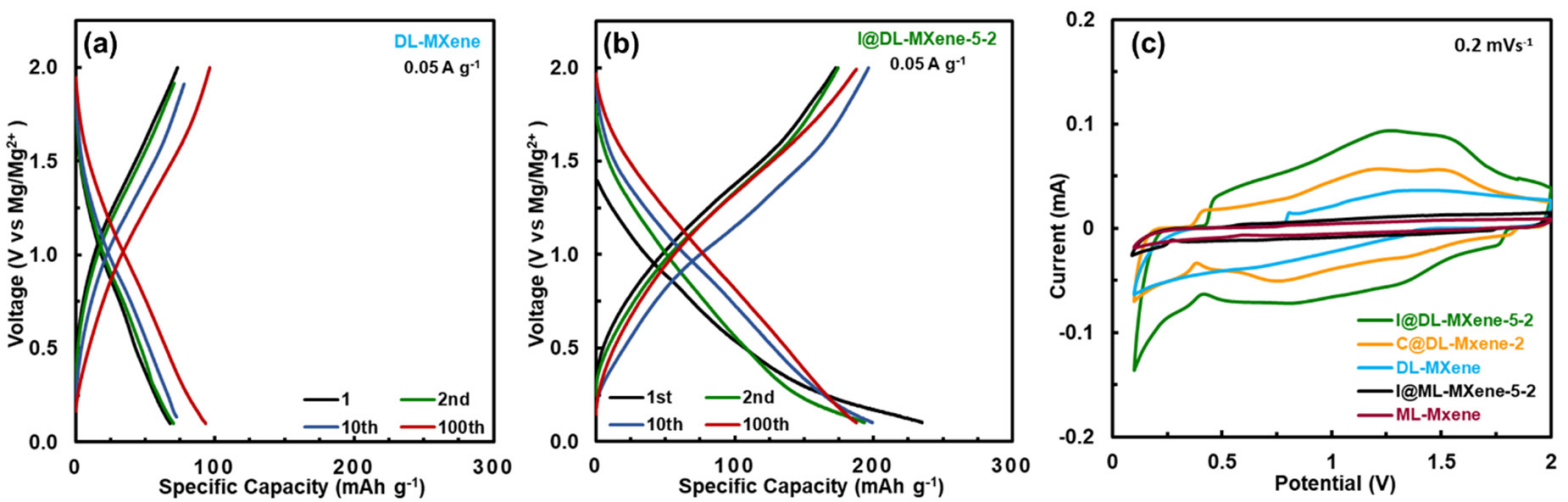
Galvanostatic charge–discharge (GCD) curves of magnesium batteries with (**a**) DL-MXene and (**b**) I@DL-MXene-5-2 cathodes at 50 mA g^−1^ and (**c**) comparison of cyclic voltammetry (CV) curves of different cathodes at 0.2 mV S^−1^. Reproduced from Ref. [42] with permission from Wiley.

I@DL-MXene-5-2 was used as the cathode material to assemble magnesium batteries with a magnesium metal anode and 0.4 M (PhMgCl)_2_+AlCl_3_ (all phenyl complex, APC) in THF as an electrolyte. As the ionomer had good compatibility with low-polarity THF due to its hyperbranched polyethylene skeleton, ionomer modification beneficially improved the wetting of the surface by the THF-based electrolyte and facilitated the fast transport of the THF-based electrolyte. On the basis of galvanostatic charge–discharge tests at 50 mA g^−1^ within a volage window of 0.05–2.0 V, I@DL-MXene-5-2 showed an initial discharge capacity of 235 mAh g^−1^ and a reversible capacity of around 200 mAh g^−1^ on the basis of the MXene mass, in sharp contrast to the significantly lower reversible capacity of 70 mAh g^−1^ for DL-MXene and the negligible capacity of only around 3 mAh g^−1^ for ML-MXene. After 100 cycles at 50 mA g^−1^, the discharge capacity of I@DL-MXene-5-2 was well maintained at 190 mAh g^−1^, with high capacity-retention of about 95%. See Figure 6. Its performance was also significantly better compared to DL-MXene intercalated with cetyltrimethylammonium bromide (CTAB), a well-defined small-molecular analog of the ionomer but with no good affinity to THF. In the rate performance tests, I@DL-MXene-5-2 showed high capacity-retention of 52% at 1000 mAh g^−1^ relative to the capacity at 20 mA g^−1^. In reference to many reported MXene-based cathodes in rechargeable Mg batteries, the overall performance of the ionomer-modified I@DL-MXene-5-2 cathode was outstanding in terms of both the capacity and capacity-retention results. This work demonstrates the impacts of interlayer spacing engineering and nanosheet surface modification with the use of hyperbranched polyethylene ionomers on the electrochemical performance of MXene materials [42].

### 4.4. 1T/2H-MoS_2_

MoS_2_ is a 2D layered material in the family of transition metal dichalcogenides (TMDs) [57]. With its unique 2D structure and properties, MoS_2_ finds application in diverse areas, including electrochemical energy storage, electrocatalysis, photocatalysis, lubrication, etc. [58]. Commonly, MoS_2_ has three crystal phases, 1T, 2H, and 3R. Natural bulk MoS_2_ primarily exists in the 2H phase, which is the most thermodynamically stable form and is semiconductive. Moreover, 2H MoS_2_ has an interlayer distance of around 0.62 nm, which is significantly larger than that in graphite. Synthetic MoS_2_, depending on the synthesis method, may show 1T and 3R phases [59]. In particular, 1T-MoS_2_ shows metallic conductivity, and its monolayer demonstrates electrical conductivity (10 to 100 S cm^−1^) that is 10^7^ times greater than that of 2H-MoS_2_. However, 1T-MoS_2_ is commonly metastable and requires strategies to achieve stabilization [58]. MoS_2_, with high content of the stable 1T phase and an enlarged interlayer distance, shows strong potential as an electrode material for the storage of large ions (like Na^+^ and K^+^) and multivalent ions (such as Mg^2+^, Zn^2+^, and Al^3+^) in rechargeable batterie [60,61].

**Scheme 6:**
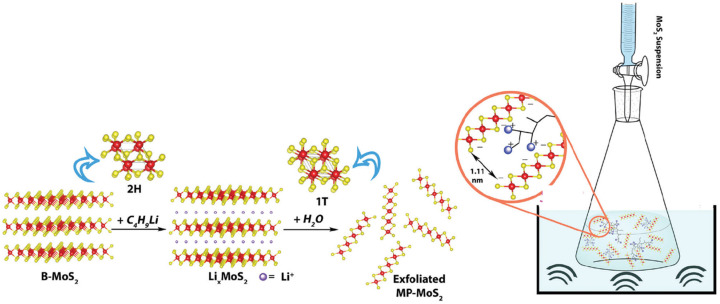
Schematic modification of 1T/2H MP-MoS_2_ with hyperbranched polyethylene ionomer. Adapted from Ref. [43] with permission from Wiley.

Our group has recently demonstrated the modification of a 1T phase-rich interlayer-expanded few-layered 1T-2H mixed-phase MoS_2_ (MP-MoS_2_) with a hyperbranched polyethylene ionomer having quaternary ammonium ion content of 1.03 mol% with BF_4_^−^ counter anion (Scheme 6) [43]. The few-layered MP-MoS_2_ was synthesized from bulk MoS_2_ (B-MoS_2_) by a top-down method, involving n-butyllithium-assisted exfoliation. In the XRD pattern of fresh MP-MoS_2_, a new downshifted (002) peak emerged at 9.65° (corresponding interlayer distance of 0.92 nm), relative to 14.37° (interlayer distance of 0.62 nm) in bulk MoS_2_, suggesting the successful formation of exfoliated 2D nanosheets intercalated with small guest molecules. However, after storage for 2 months, the new (002) peak completely disappeared due to the restacking of the exfoliated nanosheets over time. MP-MoS_2_ contained a negative surface charge of −50.4 mV and was highly hydrophilic, with its film having a water contact angle of 33°. Although stably dispersible in water, it was nearly fully precipitated after dispersion in THF for 48 h [43].

**Figure 7:**
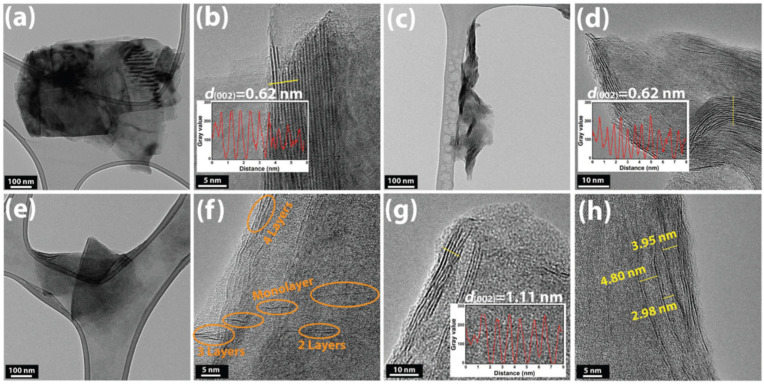
TEM images of (**a**,**b**) B-MoS_2_, (**c**,**d**) MP-MoS_2_, and (**e**–**h**) I@MP-MoS_2_. The insets in (**b**,**d**,**f**) show the intensity profiles along with the yellow lines in corresponding images. Reproduced from Ref. [43] with permission from Wiley.

The ionomer modification was conveniently performed by the dropwise addition of an aqueous dispersion of MP-MoS_2_ into the ionomer solution in THF under sonication, followed by thorough washing with fresh THF. From the thermogravimetric analysis (TGA), the ionomer-modified MP-MoS_2_ (I@MP-MoS_2_) had ionomer content of around 9% only. However, this led to dramatically changed properties. I@MP-MoS_2_ showed a positive surface charge of 27.6 mV. It also became hydrophobic, forming a stable dispersion in THF without settling within 1 week. A film of I@MP-MoS_2_ showed a hydrophobic surface with a high water contact angle of 120°. XRD showed that I@MP-MoS_2_ had a new (002) peak at 7.93° with a large interlayer distance of 1.11 nm, which was stable after 2 months of storage. TEM images of I@MP-MoS_2_ showed the presence of many few-layered clusters with interlayer distances in the range of 1.11 to 4–5 nm (see Figure 7), confirming the efficient intercalation of the ionomer within the nanosheets through the ionic interactions between the quaternary ammonium ions on the ionomer and the negatively charged nanosheet surface [43].

**Figure 8:**
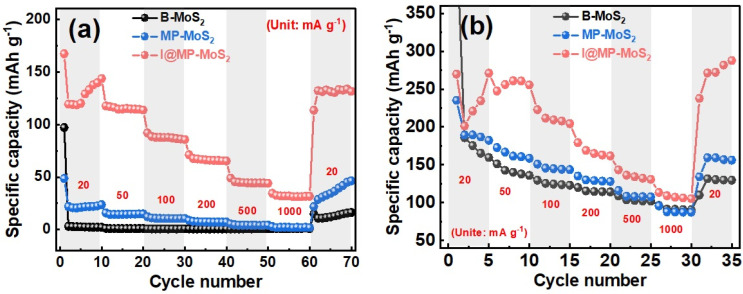
Specific capacities at different current densities for B-MoS_2_, MP-MoS_2_, and I@MP-MoS_2_ cathodes in (**a**) magnesium-ion batteries and (**b**) magnesium-/lithium-ion dual salt batteries. Adapted from Ref. [43] with permission from Wiley.

I@MP-MoS_2_ showed some distinct electrochemical properties as a cathode material for magnesium batteries with a THF-based APC electrolyte. Relative to B-MoS_2_ and MP-MoS_2_ electrodes, the I@MP-MoS_2_ electrode showed significantly lowered internal resistance during discharge–charge due to the presence of the metallic, conductive, stable 1T phase. It also demonstrated an exceptionally enhanced capacity for the storage of Mg^2+^ ions (e.g., 144 mAh g^−1^ vs. negligible capacity of about 2 mAh g^−1^ for B-MoS_2_ at 20 mA g^−1^; see Figure 8a). These improved properties were attributed to the presence of the metallic conductive stable 1T phase, the enlarged interlayer distance, and the improved electrode/electrolyte interface charge transfer as a result of the improved wettability of the nanosheet surface by the THF-based electrolyte following ionomer modification [43].

I@MP-MoS_2_ was further demonstrated as a unique cathode material in hybrid magnesium-/lithium-ion dual salt batteries (MLIBs) that contained both Mg^2+^ and Li^+^ salts in the electrolyte but with a magnesium metal anode. All three cathodes, B-MoS_2_, MP-MoS_2_, and I@MP-MoS_2_, showed similar capacities towards the storage of Li^+^. However, the former two showed much weaker capacities towards Mg^2+^ due to their small interlayer distances compared to the latter. MLIBs built with B-MoS_2_ and MP-MoS_2_ cathodes behaved as Daniell-type cells, with predominant Li^+^ insertion/extraction on the cathode side and Mg^2+^ insertion/extraction on the anode side. In those with the I@MP-MoS_2_ cathode, the co-intercalation of both Li^+^ and Mg^2+^ occurred on the cathode side, thus leading to significantly enhanced capacities (260 mAh g^−1^ vs. 161 mAh g^−1^ for MP-MoS_2_ and 140 mAh g^−1^ for B-MoS_2_ at 50 mA g^−1^; see Figure 8b). Meanwhile, it also demonstrated high capacity-retention of 87% over 200 cycles at 100 mA g^−1^ [43].

## 5. Conclusions

The development of Pd-diimine catalyst technology has enabled the synthesis of a unique range of polyethylene-based cationic ionomers that feature a hyperbranched polyethylene skeleton tethered with quaternary ammonium ions by the direct catalytic polymerization of ethylene with ionic comonomers. Controlling the polymerization conditions and feed comonomers allows, to some degree, the tuning of the ionic content and counter anion type of the ionomers. Due to the possession of the quaternary ammonium groups, despite at low contents, the ionomers show some dramatically impacted physical properties. Benefiting from their unique structures and properties, the ionomers have found some interesting and valuable applications in the surface modification of a range of one-dimensional and two-dimensional nanomaterials, including but not limited to cellulose nanocrystals, gold nanorods, Ti_3_C_2_T_x_ MXene, and MoS_2_. The ionomer-modified nanomaterials show desirable dispersibility in nonpolar or low-polarity organic phases, facilitating their processing, handling, and storage for broadened application.

Nevertheless, there are some major restrictions regarding the range of hyperbranched polyethylene ionomers developed to date. Firstly, the Pd-diimine catalyst employed for polymerization has relatively low catalytic activity and is costly, resulting in high-cost synthesis. Secondly, given the use of acrylate-type ionic comonomers, each quaternary ammonium ion in the ionomers is bonded to the hyperbranched polyethylene skeleton through a relatively weak ester linkage, which may suffer from cleavage by hydrolysis. Thirdly, the hyperbranched polyethylene skeletons of the ionomers are amorphous and mechanically weak, given their hyperbranched chain topology. Despite uniquely facilitating the surface modification of nanomaterials for their processing and applications, their broader applications in other areas, such as anion-exchange membranes for alkaline fuel cells, is restricted. The development of ionomers containing a semicrystalline polyethylene skeleton with strongly bonded cationic ions through the use of highly active polymerization catalysts is thus the direction for future research. We hope that the works summarized in this feature article serve as the impetus for future developments.

## Data Availability

No new data were created or analyzed in this study. Data sharing is not applicable to this article.
